# Socioeconomic status and risk of adenocarcinoma of the oesophagus and cancer of the gastric cardia in Scotland

**DOI:** 10.1054/bjoc.2000.1110

**Published:** 2000-07-03

**Authors:** D H Brewster, L A Fraser, P A McKinney, R J Black

**Affiliations:** Scottish Cancer Intelligence Unit, Information & Statistics Division, Trinity Park House, South Trinity Road, Edinburgh, EH5 3SQ, UK

**Keywords:** epidemiology, incidence, gastric carcinoma, oesophageal carcinoma, Scotland, socioeconomic status

## Abstract

The incidence of oesophageal adenocarcinoma and gastric cardia cancer increased strikingly in Scotland between 1977 and 1996. In contrast to other cancers of the oesophagus and stomach, which showed a clear relationship with socioeconomic status, no trend was evident for oesophageal adenocarcinoma or gastric cardia cancer during 1987–1996. © 2000 Cancer Research Campaign


*British Journal of Cancer* (2000) **83**(3), 387–390
© 2000 Cancer Research Campaign
doi: 10.1054/ bjoc.2000.1110, available online at http://www.idealibrary.com on IDEAL®

Short Communication

### Socioeconomic status and risk of adenocarcinoma of the oesophagus and cancer of the gastric cardia in Scotland

**DH Brewster, LA Fraser, PA McKinney and RJ Black**

Scottish Cancer Intelligence Unit, Information & Statistics Division, Trinity Park House, South Trinity Road, Edinburgh EH5 3SQ, UK

**Summary** The incidence of oesophageal adenocarcinoma and gastric cardia cancer increased strikingly in Scotland between 1977 and 1996. In contrast to other cancers of the oesophagus and stomach, which showed a clear relationship with socioeconomic status, no trend was evident for oesophageal adenocarcinoma or gastric cardia cancer during 1987–1996. © 2000 Cancer Research Campaign

**Keywords:** epidemiology; incidence; gastric carcinoma; oesophageal carcinoma; Scotland; socioeconomic status

The incidence of oesophageal adenocarcinoma and gastric cardia cancer is rising in many countries and the observed increases are not believed to be explained wholly by changes in diagnostic or reporting practices (Devesa and Fraumeni, 1999). The risks of cancers of the oesophagus (ICD-9 150) and stomach (151) have been correlated strongly with socioeconomic indicators, both in Scotland (Harris et al, 1998) and elsewhere (Faggiano et al, 1997). However, it has been argued that the existing international classification of oesophageal and stomach cancers is inadequate in the sense that it does not encourage distinction between cancers which may differ in aetiological terms (Dolan et al, 1999). The purpose of the present study is to provide a brief update of a previous analysis of temporal trends in the incidence of cancer of the oesophagus and stomach in Scotland (McKinney et al, 1995), focusing particularly on oesophageal adenocarcinoma and gastric cardia cancer, and to investigate the relationship of the last two with socioeconomic status.

Incident cases of cancers of the oesophagus (ICD-9 150) and stomach (151) for the 20-year period 1977–1996 were identified from the Scottish Cancer Registry. For these analyses, cancers of the oesophagus were sub-divided by histological type but since the majority of cancers of the stomach are adenocarcinomas, these were sub-divided by anatomical subsite. For the analysis of temporal trends, cases were sub-divided into seven diagnostic entities as follows:

1. Oesophagus, not microscopically verified
2. Oesophagus, morphologies other than squamous cell carcinoma or adenocarcinoma (see below)
3. Oesophagus, squamous cell carcinoma (ICD-O M-8050/3 – M-8076/3)
4. Oesophagus, adenocarcinoma (ICD-O M-8140/3 – M-8141/3, M-8190/3 – M-8231/3, M-8260/3 – M-8263/3, M-8310/3, M-8430/3, M-8480/3 – M-8490/3, M-8560/3)

*Received 4 January 2000*
*Revised 31 January 2000*
*Accepted 31 January 2000*

*Correspondence to:* DH Brewster

5. Gastric cardia (ICD-9 151.0)
6. Stomach, other specified subsites (ICD-9 151.1, 151.2, 151.3, 151.4, 151.5, 151.6)
7. Stomach, overlapping or unspecified subsites (ICD-9 151.8, 151.9).

For the analysis by socioeconomic status, these seven diagnostic entities were collapsed into four categories as follows:

a. Oesophagus, except adenocarcinoma (diagnostic entities 1, 2 and 3)
b. Oesophagus, adenocarcinoma (diagnostic entity 4)
c. Gastric cardia (diagnostic entity 5)
d. Stomach, except gastric cardia (diagnostic entities 6 and 7).

Mid-year population estimates were derived from the Annual Reports of the Registrar General for Scotland (Registrar General for Scotland, 1978–1997). Annual and quinquennial sex-specific age-standardized incidence rates were calculated for the period 1977–1996 by direct standardization to the European Standard Population (Waterhouse et al, 1976). In addition, for the total 10-year period 1987–1996, 1991 census-derived Carstairs deprivation category- (Carstairs and Morris, 1991) and sex-specific age-standardized incidence rates were calculated. The percentage changes in incidence between 1977 and 1996, were estimated by linear regression on annual log age-standardized rates. *P*-values reflect the statistical significance of the gradients of each of the fitted regression lines.

Sex-specific temporal trends in incidence rates of the seven diagnostic entities chosen for analysis are shown in Table 1. Rates of all seven diagnostic entities were, as expected, higher in males than females. Among males, between 1977 and 1996, the estimated percentage changes in incidence rates of oesophageal adenocarcinoma and gastric cardia cancer were +139.5% ($P < 0.0001$) and +94.9% ($P < 0.0001$) respectively. Corresponding estimates for females were +124.6% ($P < 0.0001$) and +65.3% ($P = 0.01$).

Age-standardized incidence rates by deprivation category and sex are shown for each of four diagnostic categories in Figures 1A–D. Although there is a clear association between deprivation and the risk of oesophageal cancers other than adenocarcinoma, and stomach cancers other than those of the gastric cardia, no trend is seen for oesophageal adenocarcinoma or gastric cardia cancer.

**Table 1** Age-standardized incidence rates of malignant neoplasms of the oesophagus and stomach by diagnostic entity and sex, during four consecutive quinquennia in Scotland, 1977–1996

| Diagnostic entity | | Age-standardized incidence rates per 100 000 person years at risk by period of diagnosis[a] | | | | Estimated % change 1977–1996[b] | Estimated absolute change 1977–1996[c] | *P*-value for trend |
|---|---|---|---|---|---|---|---|---|
| | | 1977–1981 | 1982–1986 | 1987–1991 | 1992–1996 | | | |
| Oesophagus | | | | | | | | |
| Not microscopically verified | Males | 2.5 | 2.0 | 2.1 | 2.4 | –13.0 | –0.7 | 0.39 |
| | Females | 1.4 | 1.2 | 1.1 | 1.4 | –9.4 | –0.5 | 0.42 |
| Other morphologies[d] | Males | 1.3 | 1.0 | 1.0 | 1.1 | –18.0 | –1.0 | 0.14 |
| | Females | 0.8 | 0.6 | 0.5 | 0.6 | –39.9 | –2.6 | 0.02 |
| Squamous carcinoma | Males | 4.2 | 4.8 | 5.2 | 5.4 | +42.7 | +1.9 | <0.001 |
| | Females | 3.2 | 3.3 | 4.0 | 4.4 | +57.3 | +2.4 | <0.0001 |
| Adenocarcinoma | Males | 3.9 | 4.7 | 5.5 | 8.0 | +139.5 | +4.7 | <0.0001 |
| | Females | 1.2 | 1.4 | 1.7 | 2.3 | +124.6 | +4.4 | <0.0001 |
| Stomach | | | | | | | | |
| Cardia | Males | 3.1 | 4.5 | 5.3 | 5.2 | +94.9 | +3.6 | <0.0001 |
| | Females | 1.0 | 1.3 | 1.6 | 1.4 | +65.3 | +2.7 | 0.01 |
| Other specified subsites | Males | 4.1 | 6.0 | 3.9 | 3.2 | –12.6 | –0.7 | 0.68 |
| | Females | 2.4 | 3.2 | 2.0 | 1.6 | –26.7 | –1.6 | 0.31 |
| Unspecified or overlapping subsites | Males | 24.1 | 19.1 | 18.1 | 15.2 | –43.1 | –2.9 | <0.0001 |
| | Females | 12.2 | 10.1 | 8.4 | 7.5 | –46.3 | –3.2 | <0.0001 |

[a]Rates directly age-standardized to the European standard population. [b]Percentage change estimated by fitting a linear regression line to the annual log age-standardized rates. [c]Absolute change estimated as the difference between fitted values for 1977 and 1996. [d]Morphologies other than squamous cell carcinoma or adenocarcinoma.

The incidence of oesophageal adenocarcinoma has continued to increase strikingly in Scotland in recent years. Clearly, there is scope for at least some of the increase to be an artefact of diagnostic and/or coding practice. However, a previous analysis of Scottish data suggests that the observed trend is unlikely to be due entirely to artefact (McKinney et al, 1995). Certainly, in the time period covered by the present analysis, the estimated absolute changes in incidence of oesophageal cancers other than adenocarcinoma, and of gastric cardia cancers, are insufficient to account for the estimated absolute increase in incidence of oesophageal adenocarcinoma. It is also of interest to note that rates of oesophageal adenocarcinoma and gastric cardia cancer combined have, since the early 1980s, begun to exceed rates of other malignant neoplasms of the oesophagus in males, but not in females. This sex difference, which has also been seen in the Merseyside and Cheshire area (Dolan et al, 1999), is not completely consistent with a diagnostic or coding artefact, which might be expected to have a similar effect in both males and females. However, it must be acknowledged that subsite misclassification could, at least in part, explain the observed increase in incidence of gastric cardia cancer (Ekström et al, 1999; Levi et al, 1999).

While many studies in diverse populations have found an association between socioeconomic deprivation (using a variety of measures) and risk of all cancers of the oesophagus and all cancers of the stomach (Faggiano et al, 1997), the relationship with tumour subsites and subtypes has been much less studied and results are inconsistent. Analysis of population-based cancer registration data for the West Midlands Region of the UK showed that a higher proportion of male cases of adenocarcinoma of the oesophagus and gastric cardia were classified as higher social class (based on occupation) compared both to other sites of gastric cancer, and to all sites of cancer combined (Powell and McConkey, 1990). Conversely, in the same comparison, a lower than expected proportion of cases of adenocarcinoma of the oesophagus and gastric cardia were classified as lower social class. However, in a recent population-based case–control study, Ye et al (1999) did not find any striking differences in the distribution of socioeconomic status between 90 cases of gastric cardia cancer and 1164 controls. The results of several other case–control studies have suggested an inverse association between risk of cancers arising in the vicinity of the oesophago-gastric junction and socioeconomic status in terms of education (Palli et al, 1992; Vaughan et al, 1995; Gammon et al, 1997), occupation (Palli et al, 1992; Brown et al, 1994) and income (Brown et al, 1994; Gammon et al, 1997). However, Brown et al (1994) found a non-significant increase in risk of adenocarcinoma of the oesophagus and oesophago-gastric junction with a higher level of education. Wu-Williams et al (1990) also found a non-significant increase in risk of cardia cancer with a higher level of education, although their use of neighbourhood controls may not have been ideal for studying socioeconomic differences in risk. In a hospital-based case–control study, compared to controls, Kabat et al (1993) found higher proportions of cases of adenocarcinoma of the oesophagus or cardia with higher levels of education and (in males) occupation. A prospective cohort study in The Netherlands found a lower risk both of cardia cancer and of other subsites of stomach cancer in men with the highest level of education, although there was no significant trend across the levels of educational attainment for either diagnostic entity, and the lower risk of cardia cancer was attenuated by adjustment for a variety of confounding factors in a multivariate analysis (van Loon et al, 1998). It is important to remember that all of the above studies are based on individual measures of socioeconomic status whereas the Carstairs score used in our analysis is an area (postcode sector) based indicator of deprivation based on levels of overcrowding, male unemployment, low social class and household car ownership derived from the decennial census (Carstairs and Morris, 1991). Although area-based measures are prone to misclassification and the ‘ecological fallacy’, they have been shown to be independent predictors of morbidity (Davey Smith et al, 1998) and, in certain circumstances

 *© 2000 Cancer Research Campaign*

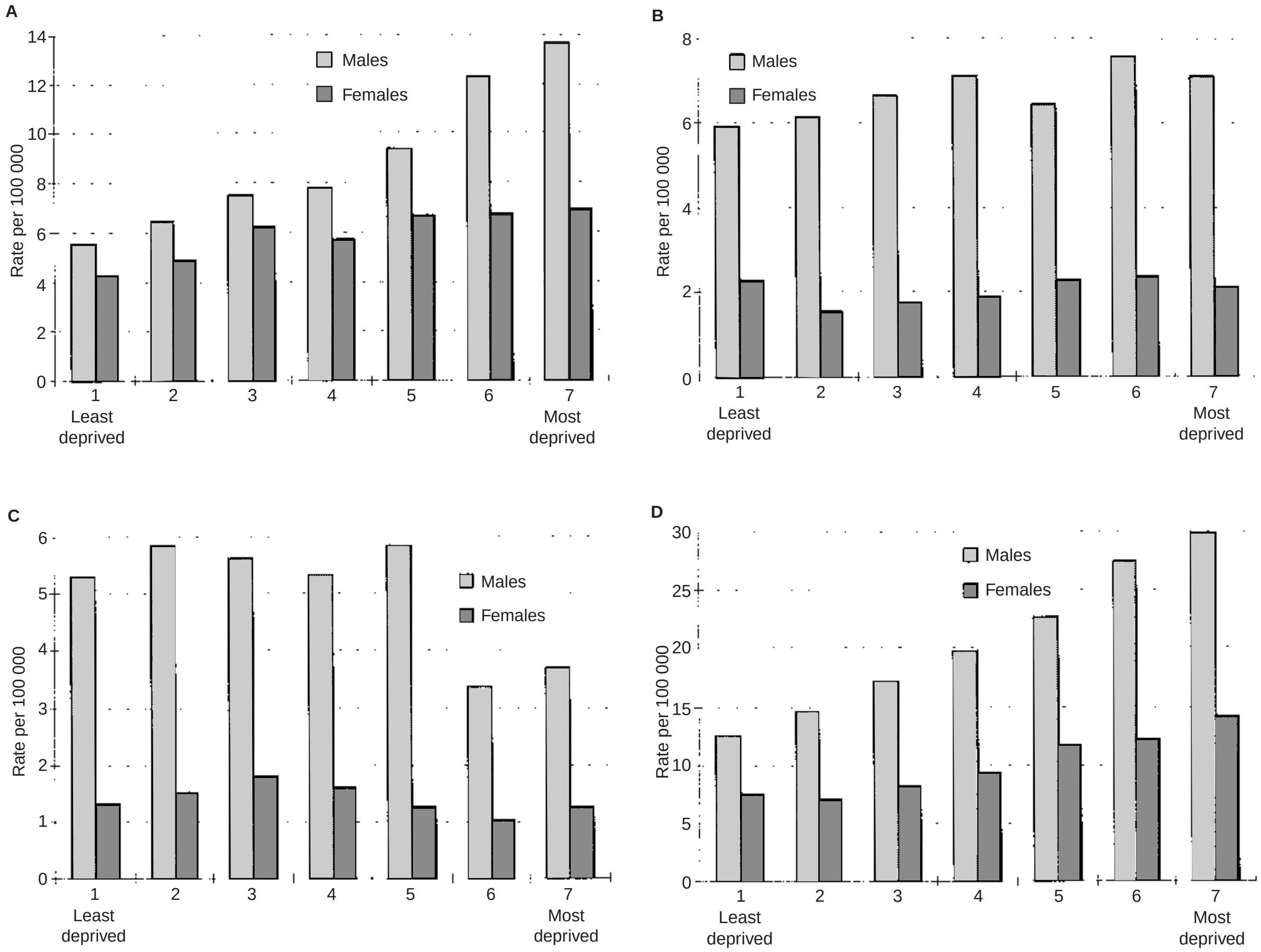

**Figure 1** Age-standardized incidence rates of malignant neoplasms of the oesophagus and stomach in Scotland by broad diagnostic category, deprivation category and sex 1987–1996. (**A**) Oesophagus, except adenocarcinoma, (**B**) oesophagus, adenocarcinoma, (**C**) gastric cardia, (**D**) stomach, except gastric cardia

(Spencer et al, 1999), may be more discriminating than individual measures of socioeconomic status.

Recent analytical studies have implicated cigarette smoking, obesity, gastro-oesophageal reflux disease and Barrett's oesophagus as the main risk factors or predisposing conditions for adenocarcinoma of the oesophagus and gastric cardia (Devesa and Fraumeni, 1999). A recent population-based case–control study in Scotland and other regions of the UK identified obesity in early adulthood and low consumption of fruit as risk factors for oesophageal adenocarcinoma in women (Cheng et al, 2000).

Given the strong inverse relationship between cigarette smoking and social class (Dong and Erens, 1997), the absence of a clear association between deprivation and risk of oesophageal adenocarcinoma and gastric cardia cancer in Scotland seems surprising. However, it is important to note that the increased risk of these cancers has been shown to persist for at least 30 years after smoking cessation (Gammon et al, 1997) so that current deprivation-category specific incidence rates may reflect smoking patterns at a time when the prevalence of smoking was distributed more evenly across the social classes in the UK (Wald and Nicolaides-Bouman, 1991). Similarly, current evidence suggests that there is not a clear relationship between obesity and social class in Scotland (Dong and Erens, 1997). Evidence is also emerging of an inverse relationship between infection with cag+ *Helicobacter pylori* strains and risk of adenocarcinoma of the oesophagus and gastric cardia (Blaser, 1999). Indeed, the declining prevalence of *H. pylori* infection has been offered as a possible explanation for the increasing incidence of adenocarcinoma of the oesophagus and gastric cardia, and the decreasing incidence of cancers of the distal stomach. If this explanation is correct, it would help to explain the tendency towards lower incidence of gastric cardia cancer among people living in more deprived communities in Scotland, since there is a well established relationship between lower social class and risk of infection with *H. pylori* in the UK (Boffetta, 1997).

In summary, in contrast to the findings for other cancers of the oesophagus and stomach, the recent striking increase in incidence of oesophageal adenocarcinoma and possibly also gastric cardia cancer, coupled with the absence of a clear association with deprivation support the view that, in Scotland as in other countries, the aetiology of these tumours is distinct.

*© 2000 Cancer Research Campaign*

### REFERENCES

Blaser MJ (1999) Hypothesis: the changing relationships of *Helicobacter pylori* and humans: implications for health and disease. *J Infect Dis* **179**: 1523–1530

Boffetta P (1997) Infection with *Helicobacter pylori* and parasites, social class and cancer. In: *Social Inequalities and Cancer*, Kogevinas M, Pearce N, Susser M and Boffetta P (eds), pp 325–329. IARC Scientific Publication No. 138. International Agency for Research on Cancer: Lyon

Brown LM, Silverman DT, Pottern LM, Schoenberg JB, Greenberg RS, Swanson GM, Liff JM, Schwartz AG, Hayes RB, Blot WJ and Hoover RNI (1994) Adenocarcinomas of the esophagus and esophagogastric junction in white men in the United States: alcohol, tobacco, and socioeconomic factors. *Cancer Causes Control* **5**: 333–340

Carstairs V and Morris R (1991) *Deprivation and Health in Scotland*. Aberdeen University Press: Aberdeen

Cheng KK, Sharp L, McKinney PA, Logan RFA, Chilvers CED, Cook-Mozaffari P, Ahmed A and Day NE (2000) The preventability of oesophageal adenocarcinoma in women: a case–control study. *Br J Cancer* (in press)

Davey Smith G, Hart C, Watt G, Hole D and Hawthorne V (1998) Individual social class, area-based deprivation, cardiovascular disease risk factors, and mortality: the Renfrew and Paisley study. *J Epidemiol Community Health* **52**: 399–405

Devesa SS and Fraumeni JF Jr (1999) The rising incidence of gastric cardia cancer. *J Natl Cancer Inst* **91**: 747–749

Dolan K, Sutton R, Walker SJ, Morris AI, Campbell F and Williams EMI (1999) New classification of oesophageal and gastric carcinomas derived from changing patterns in epidemiology. *Br J Cancer* **80**: 834–842

Dong W and Erens B (eds) (1997) *Scottish Health Survey 1995, Vol 1*. The Stationery Office: Edinburgh

Ekström AM, Signorello LB, Hansson L-E, Bergström R, Lindgren A and Nyrén O (1999) Evaluating gastric cancer misclassification: a potential explanation for the rise in cardia cancer incidence. *J Natl Cancer Inst* **91**: 786–790

Faggiano F, Partanen T, Kogevinas M and Boffetta P (1997) Socioeconomic differences in cancer incidence and mortality. In: *Social Inequalities and Cancer*, Kogevinas M, Pearce N, Susser M and Boffetta P (eds), pp 65–176. IARC Scientific Publication No. 138. International Agency for Research on Cancer: Lyon

Gammon MD, Schoenberg JB, Ahsan H, Risch HA, Vaughan TL, Chow WH, Rotterdam H, West AB, Dubrow R, Stanford JL, Mayne ST, Farrow DC, Niwa S, Blot WJ and Fraumeni JF Jr (1997) Tobacco, alcohol, and socioeconomic status and adenocarcinomas of the esophagus and gastric cardia. *J Natl Cancer Inst* **89**: 1277–1284

Harris V, Sandridge A, Black RJ, Brewster DH and Gould A (1998) *Cancer Registration Statistics Scotland, 1986–1995*. ISD Publications: Edinburgh

Kabat GC, Ng SKC and Wynder EL (1993) Tobacco, alcohol intake, and diet in relation to adenocarcinoma of the esophagus and gastric cardia. *Cancer Causes Control* **4**: 123–132

Levi F, Te V-C, Randimbison L and La Vecchia C (1999) Evaluating gastric cancer misclassification: a potential explanation for the rise in cardia cancer incidence [letter]. *J Natl Cancer Inst* **91**: 1585–1586

McKinney PA, Sharp L, MacFarlane GJ and Muir CS (1995) Oesophageal and gastric cancer in Scotland 1960–1990. *Br J Cancer* **71**: 411–415

Palli D, Bianchi S, Decarli A, Cipriani F, Avellini C, Cocco P, Falcini F, Puntoni R, Russo A, Vindigni C, Fraumeni JF Jr, Blot WJ and Buiatti E (1992) A case–control study of cancers of the gastric cardia in Italy. *Br J Cancer* **65**: 263–266

Powell J and McConkey CC (1990) Increasing incidence of adenocarcinoma of the gastric cardia and adjacent sites. *Br J Cancer* **62**: 440–443

Registrar General for Scotland (1978–97). *Annual Reports 1977–1996*. HMSO: Edinburgh

Spencer N, Bambang S, Logan S and Gill L (1999) Socioeconomic status and birth weight: comparison of an area-based measure with the Registrar General’s social class. *J Epidemiol Community Health* **53**: 495–498

van Loon AJM, Goldbohm RA and van den Brandt PA (1998) Socioeconomic status and stomach cancer incidence in men: results from The Netherlands Cohort Study. *J Epidemiol Community Health* **52**: 166–171

Vaughan TL, Davis S, Kristal A and Thomas DB (1995) Obesity, alcohol, and tobacco as risk factors for cancers of the esophagus and gastric cardia: adenocarcinoma versus squamous cell carcinoma. *Cancer Epidemiol Biomarkers Prev* **4**: 85–92

Wald N and Nicolaides-Bouman A (eds) (1991) *UK Smoking Statistics, 2nd edn*, pp 61–78. Oxford University Press: London

Waterhouse J, Muir C, Correa P and Powell J (1976) *Cancer Incidence in Five Continents, Volume III*, pp. 454–459. IARC Scientific Publication no. 15. International Agency for Research on Cancer: Lyon

Wu-Williams AH, Yu MC and Mack TM (1990) Life-style, workplace, and stomach cancer by subsite in young men of Los Angeles county. *Cancer Res* **50**: 2569–2576

Ye W, Ekström Am, Hansson L-E, Bergström R and Nyrén O (1999) Tobacco, alcohol and the risk of gastric cancer by sub-site and histologic type. *Int J Cancer* **83**: 223–229

*© 2000 Cancer Research Campaign*

